# Person-centered approach to work with drug addicts on substitution maintenance therapy

**DOI:** 10.1192/j.eurpsy.2023.1188

**Published:** 2023-07-19

**Authors:** N. Halytska-Pasichnyk, S. Moroz, V. Semenikhina

**Affiliations:** 1 Psychologic, narcologic and psychiatric departments, “Dnipropetrovsk multiprofile clinical hospital for provision of psychiatric care” Dnipropetrovsk regional council”, DNIPRO, Ukraine

## Abstract

**Introduction:**

According to the United Nations World Drug Report 2019, there are an estimated 53 million opioid users[1]. Health problems, social problems associated with this abuse are the result of a complex interaction between psychoactive substances, the patient and the environment. There are two main approaches in the treatment - aimed at complete abstinence from the use of a psychoactive substance and substitution maintenance therapy using methadone and buprenorphine [2]. This is due to the pharmacological effects of narcotic substances, the dynamics of physiological processes and the general state of health with the systematic use of psychoactive substances.

**Objectives:**

The problem of the use of narcotic substances must be considered not only as a physiological problem, but also as a problem of a person resorting to drugs in a specific social situation. In this case, curative and rehabilitation work acquires new content and new opportunities. The emergence of addiction depends on two main reasons: on the one hand, these are certain specific personality traits of a person, formed primarily by the family, and on the other hand, external factors such as the influence of friends, curiosity, life difficulties experienced and other personal problems. The greatest danger of the formation of drug addiction occurs in the case of simultaneous exposure to both groups of causes.

**Methods:**

The study interviewed 123 patients aged 23 to 45 years (109 men and 14 women) diagnosed with opioid addiction. Of these, 17 patients with a period of abuse of up to three years and 105 - longer than three years, respectively. Personality disorders were assessed using the 16-factor Cattell Personality Questionnaire for the study of personality traits [3], the multi-aspect MMPI methodology (mini-mult) [4], and the Luscher color choice method [5].

**Results:**

14 drug addicts (82%) with a period of abuse of less than 3 years were characterized by impulsivity, reduced stress resistance, risk appetite; in 83 drug addicts (79%) with a period of abuse longer than 3 years, persistent psychosocial maladjustment, communication difficulties, conflicts were detected, social norms were disregarded, increased readiness for open manifestation of aggression; 14 women (100%) had features of mental rigidity, disregard for social norms, conflict, impulsiveness; 88 men (81%) had emotional instability, excitability, hostility.

**Conclusions:**

The formation of opioid dependence is influenced by both the psychophysiological characteristics and the pathopsychological changes that occurred during the use of a psychoactive substance.

Addiction therapy should include a psychological analysis of risk factors.

Selection of psychological options for social adaptation that contribute to the maximum possible realization of the individual’s potential, will increase the effectiveness of substitution therapy and accelerate the resocialization of drug addicts.

**Disclosure of Interest:**

None Declared

